# Supplementary material for: Lipid droplets modulate proteostasis, SQST-1/SQSTM1 dynamics, and lifespan in C. elegans
Source: iScience. 2023 Sep 16;26(10):107960. doi: 10.1016/j.isci.2023.107960 (PMC10551902; doi:10.1016/j.isci.2023.107960)
Supplement: Document S1. Figures S1–S4 and Tables S3–S7 [file mmc1.pdf]

iScience, Volume *26*

Supplemental information

Lipid droplets modulate proteostasis, SQST-1/SQSTM1 dynamics, and lifespan in *C. elegans*

Anita V. Kumar, Joslyn Mills, Wesley M. Parker, Joshua A. Leitão, Diego I. Rodriguez, Sandrine E. Daigle, Celeste Ng, Rishi Patel, Joseph L. Aguilera, Joseph R. Johnson, Shi Quan Wong, and Louis R. Lapierre

Supplemental Figure 1

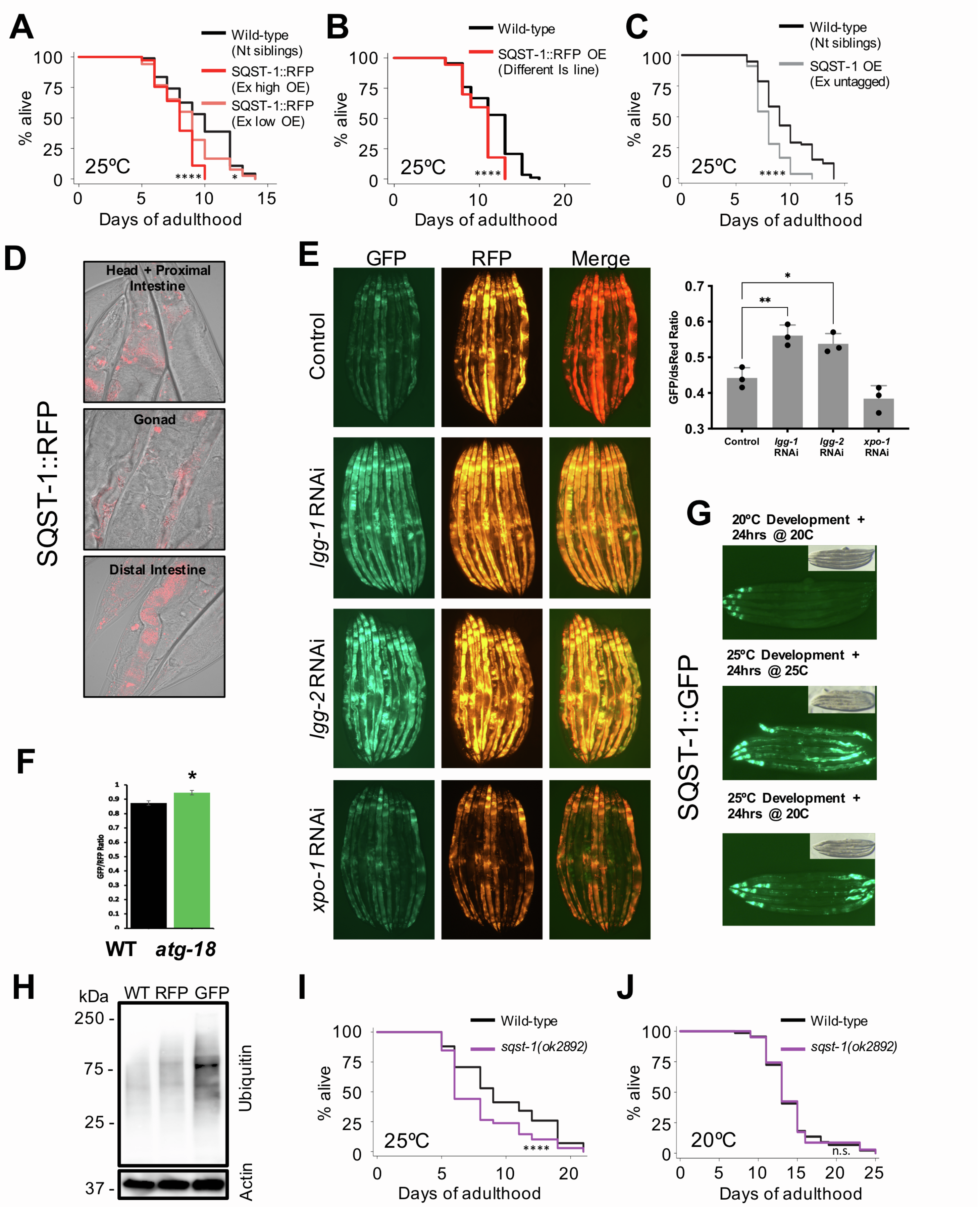

Supplemental Figure 1. The function of SQST-1 in lifespan modulation is temperature-dependent, related to Figure 1. A. Lifespan analysis of wild-type animals (non-transgenic siblings) and transgenic animals over-expressing SQST-1::RFP at low and high level (Ex: Extrachromosomal array), and **B.** transgenic animals over-expressing SQST-1::RFP (Integrated strain, Is) raised at 20ºC and grown at 25ºC during adulthood on OP50 *E. coli*. n=100. **C.** Lifespan analysis of wild-type animals (non-transgenic siblings, Nt) and transgenic animals over-expressing SQST-1 (untagged). **D.** Confocal images of SQST-1::RFP-expressing animals raised at 20ºC and grown at 25ºC for 5 days. **E.** Micrographs of animals expressing SQST-1::GFP::RFP after 72 hours of feeding during adulthood control bacteria or bacteria expressing dsRNA against autophagy genes *lgg-1* or *lgg-2,* or nuclear export protein and HLH-30 modulator *xpo-1*. **F.** Quantification of the GFP and RFP signal ratio in GFP::RFP::SQST-1 over-expressing animals in a wild-type or autophagy-defective *atg-18(gk378)* background. Average of 10 worms per image, n=3-4 images per condition *t-test* *p<0.05, **p<0.01. **G.** Images (GFP and transmitted light) of animals over-expressing SQST-1::GFP were raised at 20ºC or 25ºC and kept or transferred to 20ºC, or kept at 25ºC for 24 hours. **h.** Immunoblot of ubiquitin and actin in Day 1 wild-type (WT) animals and SQST-1::RFP (RFP) and SQST-1::GFP (GFP) over-expressing animals raised at 25ºC. Lifespan analysis of wild-type animals and *sqst-1(ok2892)* animals raised at 20ºC and grown at 25ºC (**I**) or 20ºC (**J**) during adulthood on OP50 *E. coli*. n=100. Details on lifespan analyses and repeats are available in Supplemental Table 3, Mantel-Cox log-rank. n.s.: not significant, **p<0.05,****p<0.001,*.

Supplemental Figure 2

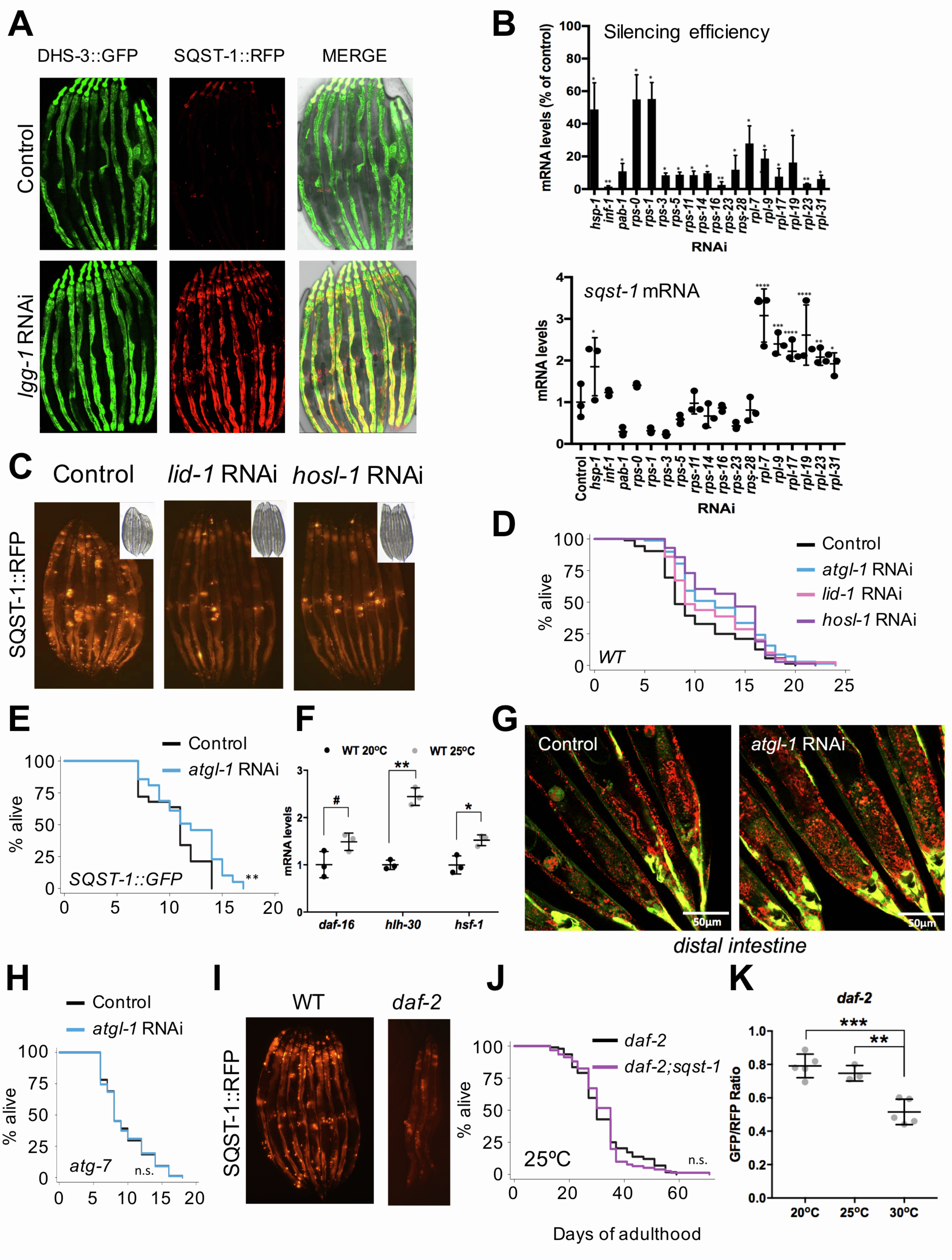

Supplemental Figure 2. Lipid droplets enhance lifespan, autophagy, and the response to heat stress, related to Figures 2 and 3. A. Image of whole animals expressing both SQST-1::RFP and the lipid droplet-resident protein DHS-3 fused to GFP, subjected to *lgg-1* silencing for 3 days during adulthood. **B.** qPCR analysis of gene silencing efficiency of each modifier and their corresponding levels of *sqst-1* mRNA after 4 days of silencing at 25ºC during adulthood of animals expressing SQST-1::RFP. n=3, ±SD, *t-test* (silencing efficiency) or *ANOVA* (*sqst-1* mRNA) *$p<0.05$, **$p<0.01$. **C.** Day 1 adult animals expressing SQST-1::RFP were fed control bacteria or bacteria expressing dsRNA against *lid-1 or hosl-1* RNAi for 96 hours and were visualized by fluorescence microscopy. **D.** Lifespan analysis of wild-type animals raised on OP50 *E. coli* at 20ºC and grown at 25ºC on control bacteria or bacteria expressing dsRNA against *atgl-1, lid-1* or *hosl-1*. Brightfield image is seen in the insert. **E.** Lifespan analysis of transgenic animals over-expressing SQST-1::GFP raised on OP50 *E. coli* at 20ºC and grown at 25ºC on control bacteria or bacteria expressing dsRNA against *atgl-1*. **F.** qPCR analysis *daf-16, hlh-30* and *hsf-1* in Day 1 wild-type animals raised at 20ºC or 25ºC on OP50 *E. coli*. Biological triplicates *t-test* #$p=0.06$, *$p<0.05$, **$p<0.01$ **G.** Representative confocal microscopy images of the distal intestine of animals expressing mCherry::GFP::LGG-1 and raised at 20ºC and fed during adulthood control bacteria or bacteria expressing dsRNA against *atgl-1* for 2 days at 25ºC. **H.** Lifespan analysis of *atg-7(bp411)* mutants raised on OP50 *E. coli* at 20ºC and grown at 25ºC on control bacteria or bacteria expressing dsRNA against *atgl-1*. **I.** Image of *daf-2(e1370)* animals over-expressing SQST-1::RFP raised on OP50 *E. coli*. bacteria at 20ºC and transferred to 25ºC during adulthood for 5 days. Comparative WT image in Figure 1A. **J.** Lifespan analysis of *daf-2(e1370)* and *daf-2(e1370);sqst-1(ok2892)* raised at 20ºC and grown at 25ºC during adulthood on OP50 *E. coli*. bacteria (n=100). **K.** Levels of GFP and RFP were measured in transgenic tandem *daf-2;SQST-1::GFP::RFP* animals after incubating Day 1 animals at 20°C, 25°C or 30°C for 24 hours on OP50 *E. coli*. Average of 10 worms per image. n=3-5 image per condition *t-test* **$p<0.01$, ***$p<0.001$. Details on lifespan analyses and repeats are available in Supplemental Tables 3 and 4, Mantel-Cox log-rank. n.s.: not significant, **$p<0.01$.

Supplemental Figure 3

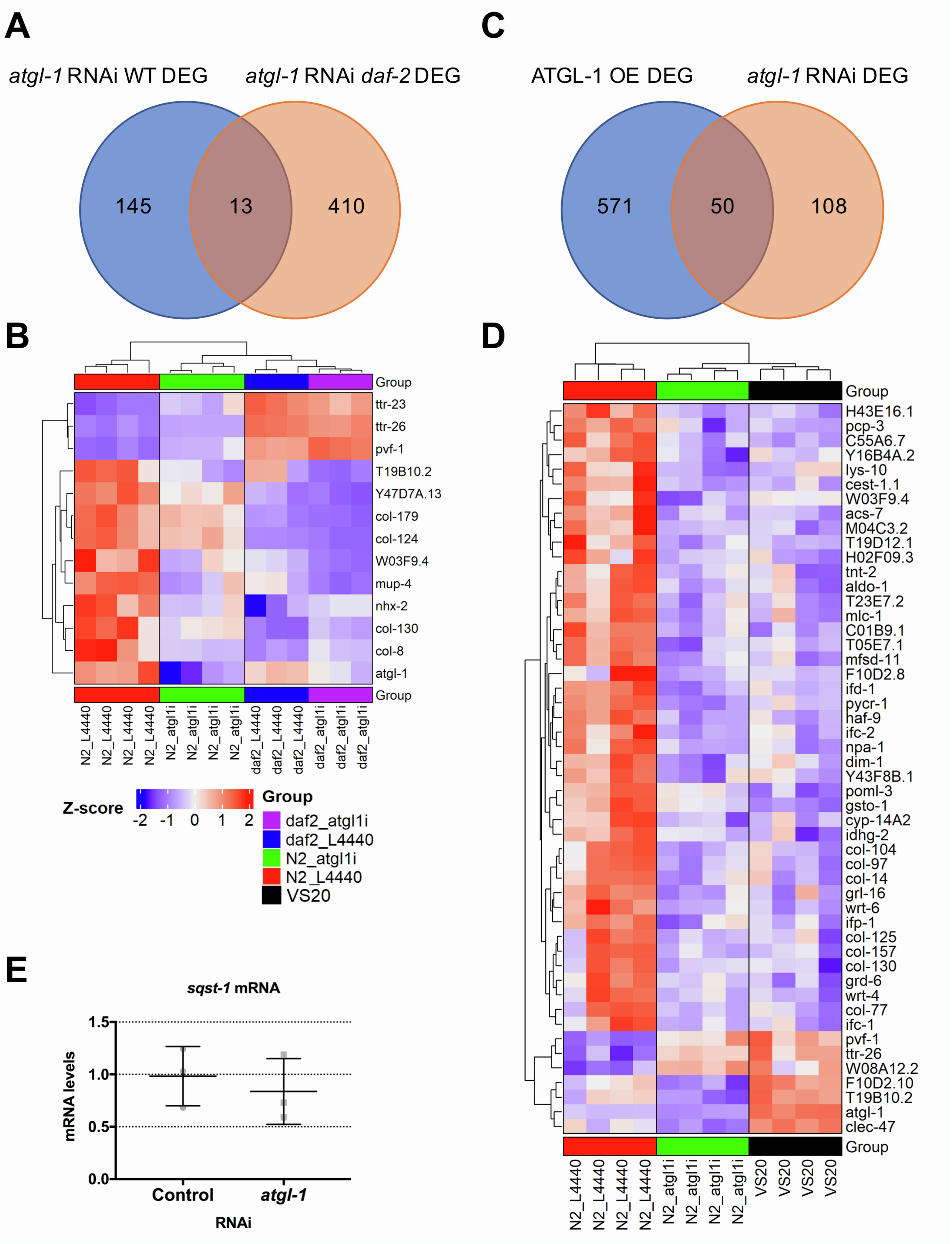

Supplemental Figure 3. Transcriptomic analyses of animals with over-expressed or silenced *atgl-1* reveal limited transcriptional changes, related to Figure 3.

A. Overlap of differentially expressed genes (DEGs) in WT and *daf-2* mutant backgrounds when *atgl-1* is silenced during adulthood at 25ºC for 4 days. **B.** Identity of overlapping DEGs from a. and their regulation status in each strain. **C.** Overlap of DEGs between nematodes over-expressing ATGL-1::GFP and adult-only *atgl-1* silencing in wild-type animals at 25ºC for 4 days. **D.** Identity of overlapping DEGs from c. and their regulation status under each condition. Heatmaps were created using Rstudio. **E.** qPCR analysis of *sqst-1* mRNA in wild-type animals fed during adulthood control bacteria or bacteria expressing dsRNA against *atgl-1* for 4 days at 25ºC. n=3 ±SD *t*-test.

Supplemental Figure 4

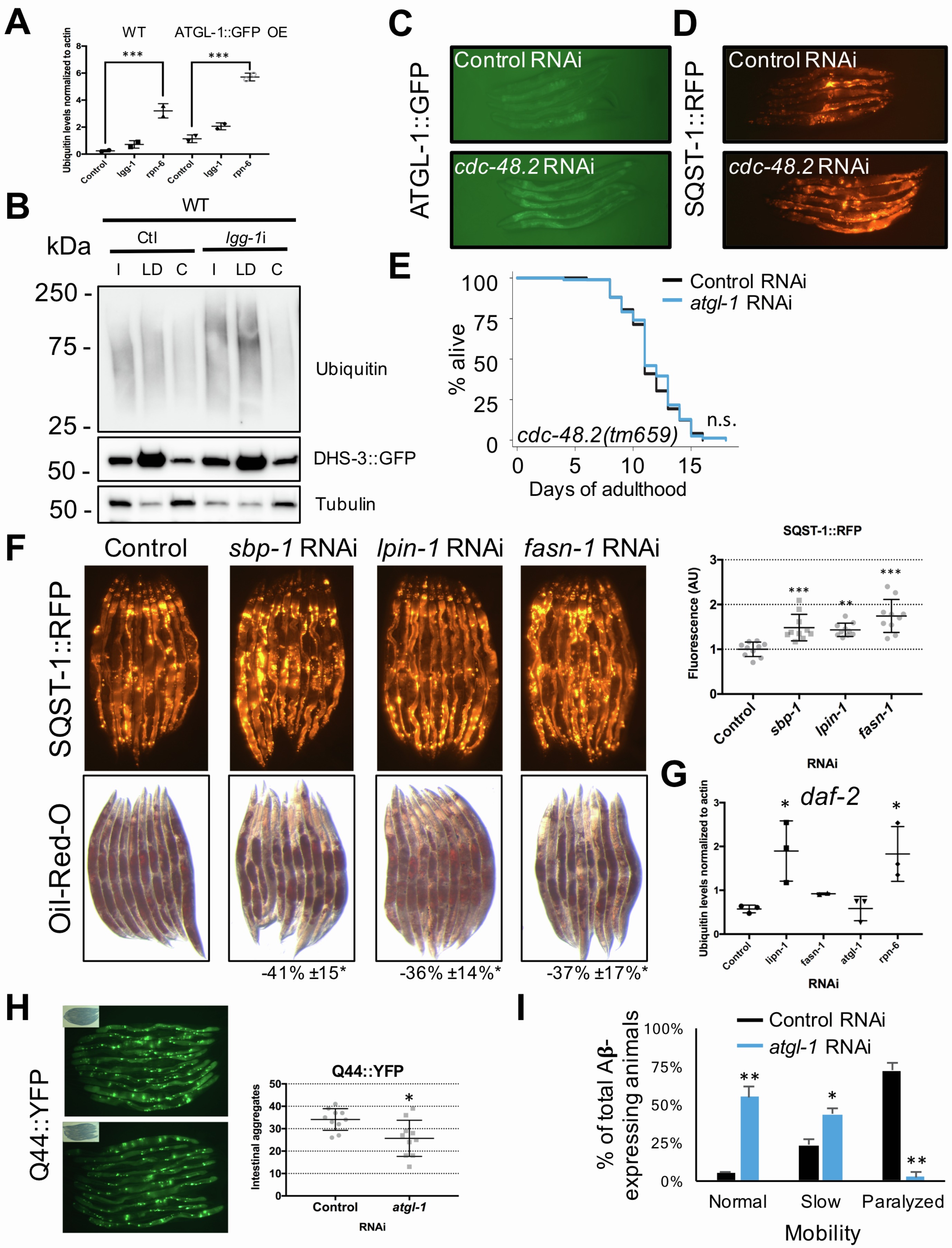

Supplemental Figure 4. Roles of lipid droplet levels in protein ubiquitination, SQST-1 dynamics and protein aggregation, related to Figure 4. A. Quantification of polyubiquitinated proteins in Figure 4C. **B.** Animals expressing lipid droplet-resident protein DHS-3 fused to GFP were raised at 20°C and then grown at 25°C during adulthood on control bacteria or bacteria expressing RNAi against *lgg-1* for 4 days. Levels of ubiquitinated proteins, DHS-3::GFP and cytosolic marker tubulin were immunoblotted from total input (I), cytosol (C) and lipid droplet (LD) fractions (fractions loaded comparatively, i.e. 10%). **C.** Day 1 ATGL-1::GFP and **D.** SQST-1::RFP over-expressing nematodes were fed control bacteria or bacteria expressing dsRNA against *cdc-48.2* for 2 days at 25ºC. **E.** Lifespan analysis of *cdc-48.2* mutants fed control bacteria or bacteria expressing dsRNA against *atgl-1* during adulthood at 25ºC. **F.** Day 1 SQST-1::RFP over-expressing nematodes were fed control bacteria or bacteria expressing dsRNA against *sbp-1, lpin-1,* or *fasn-1* for 6 days at 25ºC. Corresponding images of nematodes stained with Oil-Red-O. Fluorescence was quantified using Image J. ±SD *ANOVA* $^*p<0.05$, $^{****}p<0.001$. **G.** Quantification of polyubiquitinated protein levels in Figure 4G. **H.** Nematodes expressing intestinally Q44::YFP were grown at at 20°C on OP50 *E. coli* and fed control bacteria or bacteria expressing dsRNA against *atgl-1* at Day 1 of adulthood for 4 days at 25°C, followed by quantification of intestinal aggregates. ±SD *t*-test $^*p<0.05$. **I.** Nematodes expressing heat-inducible human A**β**-42 were grown at 20°C on OP50 *E. coli* and fed control bacteria or bacteria expressing dsRNA against *atgl-1* at Day 1 of adulthood for 2 days at 25°C. Paralysis was scored thereafter. Triplicates of n=100 each, ±SD *t*-test $^*p<0.05$, $^{**}p<0.01$. Details on lifespan analyses and repeats are available in Supplemental Table 4, Mantel-Cox log-rank. n.s.: not significant.

Supplemental Table 1

See Supplemental Table 1 Excel File

Supplemental Table 2

See Supplemental Table 2 Excel File

Supplemental Table 3

Strains	Transgenic Mean Lifespan	Events Observed	N2 Control Mean Lifespan	Events Observed	% Difference	P Value	Fig.
**25ºC**							
LRL160 (llcIs12 (sqst-1p::sqst-1::RFP::unc-54 + myo-2p::GFP))	11.9	43/100	13.5	50/100	-11%	0.0661	
LRL160 (llcIs12 (sqst-1p::sqst-1::RFP::unc-54 + myo-2p::GFP))	10.5	76/100	11.5	87/100	-9%	0.0094	
LRL160 (llcIs12 (sqst-1p::sqst-1::RFP::unc-54 + myo-2p::GFP))	9.8	57/100	12.9	49/100	-24%	<0.0001	1A
LRL161 (llcIs13 (sqst-1p::sqst-1::RFP::unc-54 + myo-2p::GFP))	10.1	84/100	11.5	87/100	-12%	<0.0001	S1B
MAH349(sqIs35[pMH951/SQST::GFP/p62::GFP+pMH876/unc-122p::rfp])	7.7	50/100	9.9	53/100	-22%	<0.0001	
MAH349(sqIs35[pMH951/SQST::GFP/p62::GFP+pMH876/unc-122p::rfp])	7.9	48/100	10.0	86/100	-21%	<0.0001	
MAH349(sqIs35[pMH951/SQST::GFP/p62::GFP+pMH876/unc-122p::rfp])	7.9	58/100	10.6	71/100	-26%	<0.0001	1B
MAH349(sqIs35[pMH951/SQST::GFP/p62::GFP+pMH876/unc-122p::rfp])	9.6	28/100	11.0	48/100	-13%	0.0320	
LRL132 (llcEx55 (sqst-1p::sqst-1::GFP::RFP::unc-54))	10.7	89/100	11.7	74/100	-9%	0.0017	1B
LRL132 (llcEx55 (sqst-1p::sqst-1::GFP::RFP::unc-54))	10.0	89/100	10.9	70/100	-8%	0.0030	
LRL90(llcEx39 (sqst-1p::sqst-1::RFP::unc-54 + myo-2p::GFP)) Ex high	7.9	124/160	9.6	57/100	-18%	<0.0001	S1A
LRL90(llcEx39 (sqst-1p::sqst-1::RFP::unc-54 + myo-2p::GFP)) Ex low	8.6	79/100	9.6	57/100	-11%	0.0250	S1A
LRL90(llcEx39 (sqst-1p::sqst-1::RFP::unc-54 + myo-2p::GFP)) Ex high	9.8	87/100	11.2	75/100	-13%	0.0061	
LRL90(llcEx39 (sqst-1p::sqst-1::RFP::unc-54 + myo-2p::GFP)) Ex low	9.5	69/100	11.2	75/100	-15%	0.0021	
MAH844 (Exsq146 (sqst-1p::sqst-1 + rol-6))	8.0	61/100	9.6	68/100	-16%	<0.0001	S1C
VC2196 (sqst-1 (ok2892) IV)	7.5	87/100	9.9	53/100	-24%	<0.0001	
VC2196 (sqst-1 (ok2892) IV)	7.9	81/100	10.0	86/100	-21%	<0.0001	S1I
VC2196 (sqst-1 (ok2892) IV)	9.3	75/100	10.6	71/100	-12%	0.0420	
VC2196 (sqst-1 (ok2892) IV)	9.5	63/100	11.0	48/100	-14%	0.0023	
VS20 (hjIs67 [atgl-1p::atgl-1::GFP + mec-7::RFP])	11.8	74/100	13.5	50/100	-12%	0.0203	4A
VS20 (hjIs67 [atgl-1p::atgl-1::GFP + mec-7::RFP])	10.1	59/100	10.7	79/100	-6%	0.0312	
VS20 (hjIs67 [atgl-1p::atgl-1::GFP + mec-7::RFP])	11.6	61/100	12.9	49/100	-10%	0.0035	
LRL24 (daf-2(e1370) III; sqIs35[pMH951/SQST::GFP/p62::GFP+pMH876/unc-122p::rfp])	30.7	95/100	27.9 (*daf-2*)	95/100	10%	0.1258	
LRL24 (daf-2(e1370) III; sqIs35[pMH951/SQST::GFP/p62::GFP+pMH876/unc-122p::rfp])	31.9	92/100	29.3 (*daf-2*)	89/100	9%	0.5998	3J
LRL170 (daf-2(e1370) III; sqst-1 (ok2892) IV)	32.0	89/100	32.5 (*daf-2*)	80/100	-2%	0.8749	S2E
**20ºC**							
LRL160 (llcIs12 (sqst-1p::sqst-1::RFP::unc-54 + myo-2p::GFP))	14.0	62/100	15.1	68/100	-7%	0.1021	1D
LRL160 (llcIs12 (sqst-1p::sqst-1::RFP::unc-54 + myo-2p::GFP))	15.3	48/100	16.3	50/100	-6%	0.2725	
LRL161 (llcIs13 (sqst-1p::sqst-1::RFP::unc-54 + myo-2p::GFP))	14.0	63/100	15.1	68/100	-7%	0.0904	
MAH349(sqIs35[pMH951/SQST::GFP/p62::GFP+pMH876/unc-122p::rfp])	14.1	42/100	14.0	49/100	1%	0.6271	1D
MAH349(sqIs35[pMH951/SQST::GFP/p62::GFP+pMH876/unc-122p::rfp])	15.3	48/100	14.2	30/100	7%	0.3137	
LRL132 (llcEx55 (sqst-1p::sqst-1::GFP::RFP::unc-54))	14.1	68/100	15.0	42/100	-6%	0.1409	1F
VC2196 (sqst-1 (ok2892) IV)	14.1	52/100	14.0	49/100	1%	0.8730	S1J
VS20 (hjIs67 [atgl-1p::atgl-1::GFP + mec-7::RFP])	15.2	77/100	11.9	58/100	27%	<0.0001	

Supplemental Table 3. Lifespan analyses performed on OP50 *E. coli*. Animals were raised at 20ºC and grown at 20ºC or 25ºC (as noted) during adulthood on OP50 *E. coli*. Mantel-Cox log-rank.

Supplemental Table 4

Strains	*atgl-1* RNAi Mean Lifespan	Events Observed	Control RNAi Mean Lifespan	Events Observed	% Difference	P Value	Fig.
N2	16.0	59/100	13.1	53/100	22%	0.0017	3A
N2	11.9	75/100	10.4	79/100	14%	0.0019	
N2	12.6	90/100	10.6	79/100	19%	<0.0001	
N2	12.7	66/100	9.9	50/100	28%	<0.0001	
N2	11.4	52/100	10.2	60/100	12%	0.0461	
N2	11.5	64/100	10.1	72/100	14%	0.0009	
N2	11.8	57/100	9.9	67/100	19%	0.0001	
N2	13.1	75/100	11.1	53/100	18%	0.0231	
N2	12.7	62/100	10.5	61/100	21%	<0.0001	
N2	12.8	55/100	11.7	65/100	9%	0.0468	
N2	11.3	66/100	9.1	85/100	24%	0.0003	
N2	12.5	74/100	10.1	76/100	24%	0.0013	S2D
CF1037 (daf-16 (mu86) I)	14.0	55/100	11.6	53/100	21% (22)	0.0010	3E
CF1037 (daf-16 (mu86) I)	10.1	83/100	9.0	81/100	12% (14)	0.0002	
LRL31 (hlh-30 (tm1978) IV)	14.7	72/100	12.1	62/100	22% (22)	<0.0001	3F
LRL31 (hlh-30 (tm1978) IV)	8.7	59/100	7.9	77/100	10% (14)	<0.0001	
CF2495 (hsf-1 (sy441) I)	12.4	36/100	11.9	35/100	4% (22)	0.5855	3G
CF2495 (hsf-1 (sy441) I)	10.7	86/100	10.2	85/100	5% (14)	0.3276	
MAH349 (sqls35[sqst-1p::sqst-1::GFP + unc-122p::rfp])	11.8	40/100	9.8	44/100	20%(19)	0.0010	S2D
LRL160 (llcls12 (sqst-1p::sqst-1::RFP::unc-54 + myo-2p::GFP))	12.1	73/100	8.6	69/100	41% (19)	<0.0001	3C
HZ1686 (atg-7 bp411 IV)	9.5	85/100	9.5	82/100	0%(14)	0.9579	S2G
HZ1686 (atg-7 bp411 IV)	9.0	73/100	9.3	58/100	-3% (19)	0.5323	
CF1041 (daf-2 e1370 III)	35.0	80/100	27.3	76/100	28% (18)	<0.0001	3J
CF1041 (daf-2 e1370 III)	25.1	60/100	23.0	81/100	9% (18)	0.0312	
FX544 (cdc-48.1 (tm544) II)	12.4	79/100	12.3	77/100	-1% (21)	0.7901	
FX659 (cdc-48.2 (tm659) II)	9.4	47/100	9.5	54/100	1% (12)	0.8290	S4D
FX659 (cdc-48.2 (tm659) II)	11.6	79/100	11.5	76/100	1% (21)	0.5981	
**Strains**	***lid-1* RNAi Mean Lifespan**	**Events Observed**	**Control RNAi Mean Lifespan**	**Events Observed**	**% Difference**	**P value**	
N2	10.8	62/100	9.1	85/100	19%	0.0080	
N2	11.7	60/100	10.1	76/100	16%	0.0510	S2D
**Strains**	***hosl-1* RNAi Mean Lifespan**	**Events Observed**	**Control RNAi Mean Lifespan**	**Events Observed**	**% Difference**	**P value**	
N2	12.3	69/100	9.1	85/100	35%	<0.0001	
N2	13.1	79/100	10.1	76/100	30%	0.0003	S2D
**Strains**	***lipn-1* RNAi Mean Lifespan**	**Events Observed**	**Control RNAi Mean Lifespan**	**Events Observed**	**% Difference**	**P value**	
N2	11.4	79/100	12.1	74/100	-6%	0.0982	
N2	10.2	78/100	11.1	80/100	-8%	0.0475	4H
CF1041 (daf-2 e1370 III)	26.1	89/100	32.3	56/100	-19%	0.0003	
CF1041 (daf-2 e1370 III)	26.4	88/100	33.4	57/100	-21%	<0.0001	4H
**Strains**	***fasn-1* RNAi Mean Lifespan**	**Events Observed**	**Control RNAi Mean Lifespan**	**Events Observed**	**% Difference**	**P value**	
N2	10.1	82/100	12.1	74/100	-16%	0.0001	
N2	10.1	69/100	11.1	80/100	-9%	0.0171	
CF1041 (daf-2 e1370 III)	26.1	77/100	32.3	56/100	-19%	0.0006	
CF1041 (daf-2 e1370 III)	29.4	83/100	33.4	57/100	-12%	0.0020	

Supplemental Table 4. Lifespan analyses related to gene silencing. Animals were developed at 20ºC on OP50 and transferred on control bacteria or bacteria expressing dsRNA against *atgl-1* and grown at 25ºC during adulthood. Comparable N2 % difference in brackets. Mantel-Cox log-rank.

Supplemental Table 5

Published strains used in this study:		
**Strain**	**Genotype**	**Strain origin**
**N2**	Wild-type, WT	Hansen Lab
**CF1037**	*daf-16 (mu86) I*	Hansen Lab
**CF1041**	*daf-2(e1370) III*	Hansen Lab
**CF2495**	*hsf-1(sy441) I*	Hansen Lab
**FX544**	*cdc-48.1(tm544) II*	CGC, National Bioresource Project at the Tokyo Women's Medical University School of Medicine
**FX659**	*cdc-48.2(tm659) II*	CGC, National Bioresource Project at the Tokyo Women's Medical University School of Medicine
**FX1978**	*hlh-30 (tm1978) IV*	CGC, National Bioresource Project at the Tokyo Women's Medical University School of Medicine
**GF80**	dgEx80 [(pAMS66) vha-6p::Q44::YFP + rol-6(su1006) + pBluescript II].	CGC
**GMC101**	dvIs100 [unc-54p::A-beta-1-42::unc-54 3'-UTR + mtl-2p::GFP]	CGC
**LIU1**	*ldrIs1 (dhs-3p::dhs-3::GFP + unc-76(+))*	CGC
**LRL9**	*atg-7 (bp411) IV*	Lapierre Lab
**MAH78**	*sqIs2[Plipl-4::LIPL-4(K04A8.5)::SL2gfp + Pmyo-2::CHERRY]*	Hansen Lab
**MAH215**	*sqIs15[lgg-1p::mCherry::GFP::lgg-1 + rol-6]*	Hansen Lab
**MAH349**	*sqIs35[sqst-1p::sqst-1::GFP + unc-122p::rfp]*	Hansen Lab
**MAH844**	*sqEx146[sqst-1p::sqst-1 + rol-6]*	CGC
**VC2196**	*sqst-1 (ok2892) IV*	CGC
**VS20**	*hjIs67 [atgl-1p::atgl-1::GFP + mec-7::RFP]*	CGC

Supplemental Table 5. Published strains used in the study

Supplemental Table 6

**New strains created for this study:**		
**Strain**	**Genotype**	**Comments**
**LRL24**	*daf-2(e1370) III; sqIs35[pMH951/SQST ::GFP/p62::GFP+pMH876/unc-122p::rfp]*	CF1041 x MAH349
**LRL31**	*hlh-30 (tm1978) IV*	FX1978 4x backcrossed to N2
**LRL90**	*llcEx39 (sqst-1p::sqst-1::RFP::unc-54 + myo-2p::GFP)*	Injected N2 with 20 ng/uL pLAP26 and 5 ng/uL pLP7. pLP7 (*myo-2p::GFP::unc-54*) was obtained from Addgene (pBCN27). pLAP26 *(sqst-1p::sqst-1::RFP::unc-54)* was generated by NEBuilder® HiFi DNA Assembly Cloning Kit (New England BioLabs Inc., Ipswich, MA) by replacing the *3XFLAG*-tag with the *RFP* sequence in pLP25 *(sqst-1p::sqst-1::3XFLAG::unc-54)* (unpublished). The *RFP* insert was PCR-amplified from Addgene plasmid #8938 (*unc-122p::RFP)* with HiFi-compatible forward primer LC209 (5' GATGTGTCTTCAGGCGCTTCTTCACGGCTCGGGCTCGATGGTGCGCTCCTCCAAGAACG 3') and reverse primer LC210 (5' GACACCAGACAAGTTGGTAATGGCTACAGGAACAGGTGGTGGC 3'). For linearizing and amplifying linear fragments of the vector backbone *sqst-1p::sqst-1::unc-54* from pLAP25, HiFi-compatible forward primer LC207 (5' CCATTACCAACTTGTCTGGTGTC 3') and reverse primer LC208 (5' GTGAAGAAGCGCCTGAAGACACATC 3') were used. Ligation of insert to the vector backbone and subsequent transformation of products into competent *E. coli*, were performed according to manufacturer's instructions.
**LRL132**	*llcEx55 (sqst-1p::sqst-1::GFP::RFP::unc-54)*	Injected N2 with 20 ng/uL pLAP29 *(sqst-1p::sqst-1::GFP::RFP::unc-54).* pLAP29 was generated by NEBuilder® HiFi DNA Assembly Cloning Kit (New England BioLabs Inc., Ipswich, MA) by inserting the *GFP* sequence 3' and 5' of the *sqst-1* and *RFP* sequences in pLAP26 *(sqst-1p::sqst-1::RFP::unc-54)* respectively. The *GFP* insert was PCR-amplified from plasmid #836 kindly provided by Dr Andrew Dillin (UC Berkeley) with HiFi-compatible forward primer LC251 (5' TTCAGGCGCTTCTTCACGGCTCGGGCTCGATGAGTAAAGGAGAAGAACTTTTC 3') and reverse primer LC252 (5' GACGTTCTTGGAGGAGCGCACCATCGAGCCGCCGCCGCCTTTGTATAGTTCATCCATGCCATG 3'). For linearizing and amplifying the vector backbone pLP26, HiFi-compatible forward primer LC244 (5' GATGGTGCGCTCCTCCAAGAACGTC 3') and reverse primer LC250 (5' GCCGTGAAGAAGCGCCTGAAGACAC 3') were used. Ligation of insert to the vector backbone and subsequent transformation of products into competent *E. coli*, were performed according to manufacturer's instructions.
**LRL160**	*llcIs12 (sqst-1p::sqst-1::RFP::unc-54 + myo-2p::GFP)*	UV irradiation of LRL90, 6x backcrossed to N2
**LRL161**	*llcIs13 (sqst-1p::sqst-1::RFP::unc-54 + myo-2p::GFP)*	UV irradiation of LRL90, 6x backcrossed to N2
**LRL170**	*daf-2(e1370) III; sqst-1 (ok2892) IV*	CF1041 x VC2196
**LRL171**	*daf-2(e1370) III; llcIs12 (p62p::p62::RFP ::unc-54 + myo-2p::GFP)*	CF1041 x LRL160
**LRL175**	*ldrIs1 (dhs-3p::dhs-3::GFP + unc-76(+)); llcIs12 (sqst-1p::sqst-1::RFP::unc-54 + myo-2p::GFP)*	LRL87 (LIU1 4x backcrossed into N2) x LRL160
**LRL178**	*hjIs67 [atgl-1p::atgl-1::GFP + mec-7::RFP]; llcIs12 (sqst-1p::sqst-1::RFP::unc-54 + myo-2p::GFP)*	VS20 x LRL160

Supplemental Table 6. New strains created for this study

Supplemental Table 7

Genes	Direction	Sequence
act-1	Forward	CTACGAACTTCCTGACGGACAAG
act-1	Reverse	CCGGCGGACTCCATACC
atgl-1	Forward	CCGGACGTCTGGTTATCTCG
atgl-1	Reverse	GCTCGTCGTAGATTGGCTGA
cyn-1	Forward	GTGTCACCATGGAGTTGTTC
cyn-1	Reverse	TCCGTAGATTGATTCACCAC
daf-16	Forward	ATCCAATTGTGCCAAGCACTAA
daf-16	Reverse	CCACCATTTTGATAGTTTCCATAGG
hlh-30	Forward	CTCATCGGCCGGCGCTCATC
hlh-30	Reverse	AGAACGCGATGCGTGGTGGG
hsf-1	Forward	GCGGCTCCGTATAAGAATGCGACTAGGC
hsf-1	Reverse	TTAAACCAAATTAGGATCCGATGGACTTGGAGTAC
hsp-1	Forward	CGCTCAGACCTTCACAACCT
hsp-1	Reverse	TGGAAAGACGTCCCTTGTCG
inf-1	Forward	CGGCTTGATCGAGGGAAACTA
inf-1	Reverse	GCCATAACGAGAGCTTGCAC
rpl-7	Forward	TCAAGCGCAGAAAGCAGAGA
rpl-7	Reverse	ACGAAGACGGAGGATCTGGA
rpl-9	Forward	ACCTTCACCGTCAAGAACCG
rpl-9	Reverse	GAAGTGGGACACGACGAACG
rpl-17	Forward	CGGAAAACAGCACCAAGTCG
rpl-17	Reverse	GATCGAGGAGGAAGTCAGCG
rpl-19	Forward	GTTTGGCTTCGGCCGTATTG
rpl-19	Reverse	AGAGCTCGTGGTAAAGGTGC
rpl-23	Forward	CAACACCGGAGCCAAGAACTTG
rpl-23	Reverse	CGTTAGCAGCGATTCTTGGC
rps-0	Forward	GATGTCGTCGTTGTTTCGGC
rps-0	Reverse	CGGGAGATCTTTCCACGGAG
rps-3	Forward	GGCTGCCAATCAAAACGTGA
rps-3	Reverse	AACCTTCTCGGCGTAGAGCT
rps-5	Forward	GGCCGATAACTGGGGATCTG
rps-5	Reverse	GCTTCTTTCCGTTGTTGCGT
rps-11	Forward	CATTCGTGAGGTCGGACTCG
rps-11	Reverse	AAGCCCTTCTTGGAGGTTCC
rps-14	Forward	GGAATGAAGGTCAAGGCCGA
rps-14	Reverse	GCGACCTCCCTTTCTTCTGG
rps-16	Forward	GGTCGCCCACTTGAGTTCTT
rps-16	Reverse	TCCTGGTCCACCGAACTTCT
rps-23	Forward	GGAAAGCCGAAGGGACTCTG
rps-23	Reverse	GTCCGAAACCAGATACGAGCAC
rps-28	Forward	AGCTTACTCTTGCTCGTGTCA
rps-28	Reverse	AGTCTTCTGGCTTCTCTCTCAG
sqst-1	Forward	TGGCTGCTGCATCATCCGCT
sqst-1	Reverse	TCAATCGTGCCGAGACCGGG

Supplemental Table 7. qPCR primers sequences
